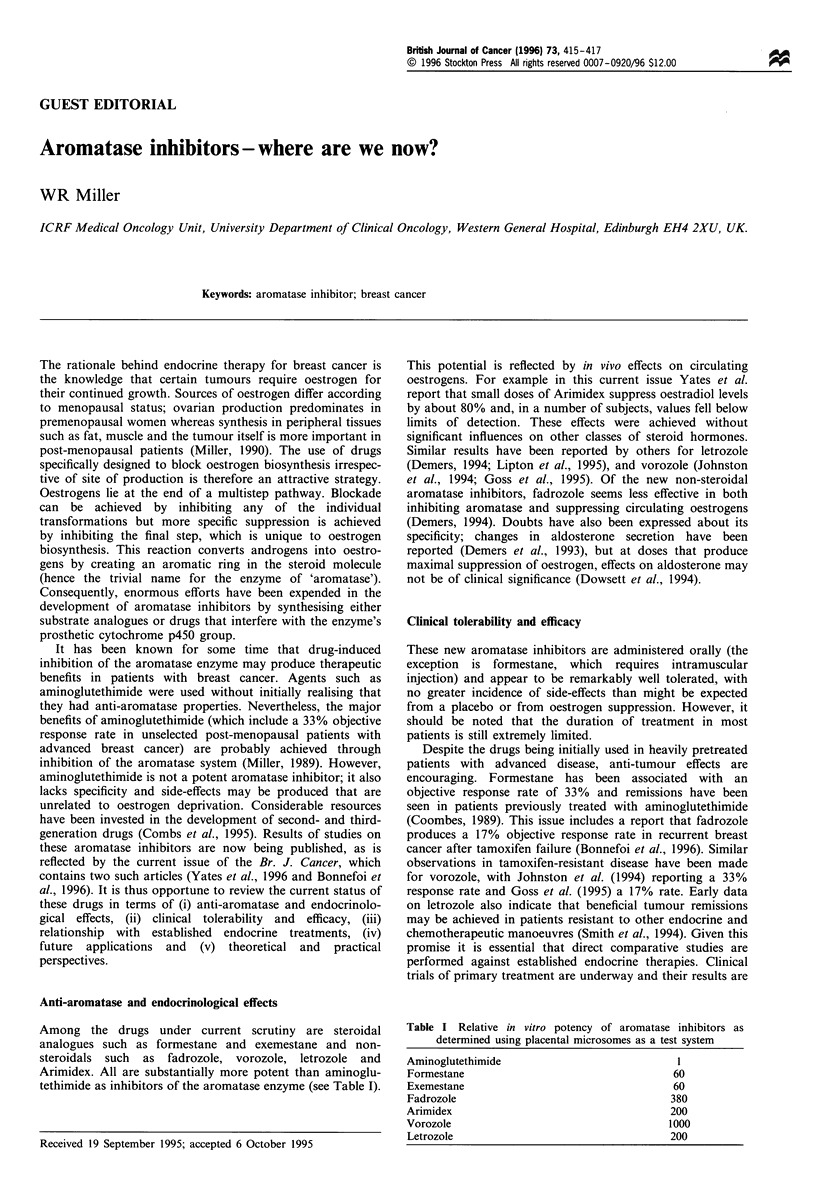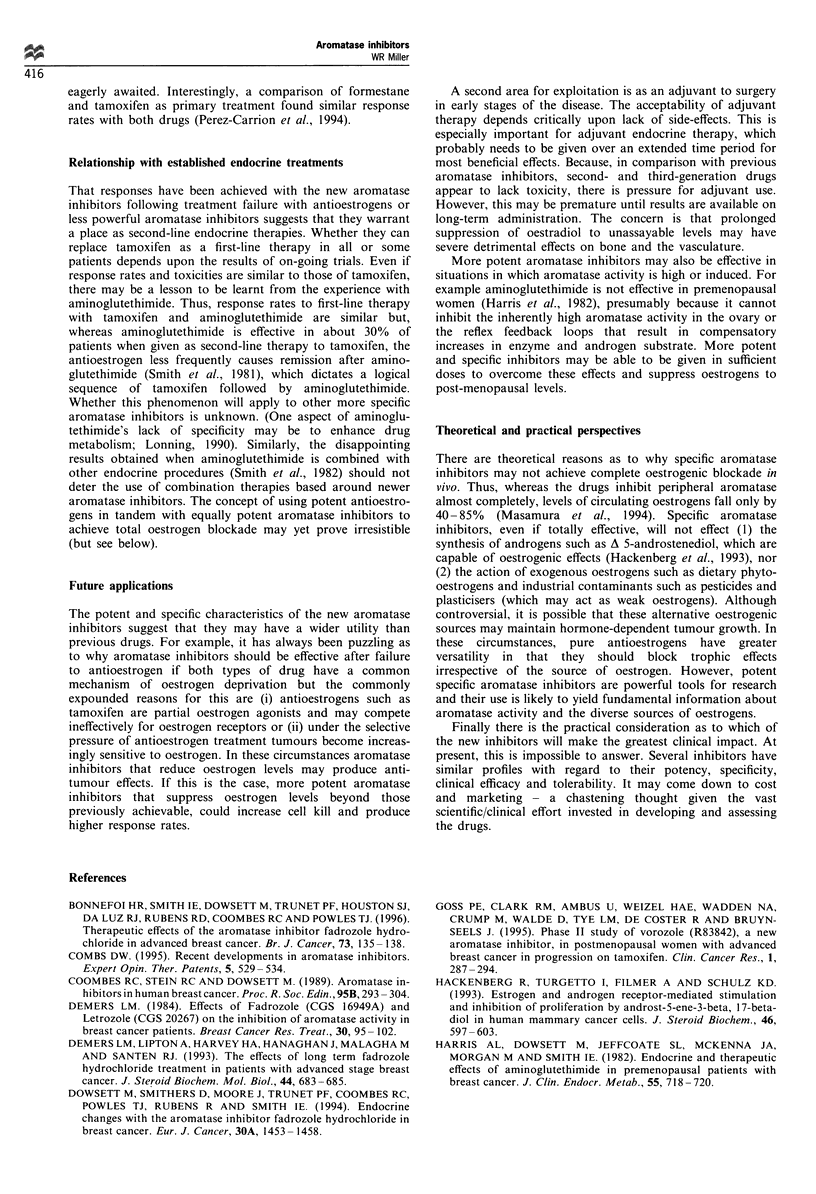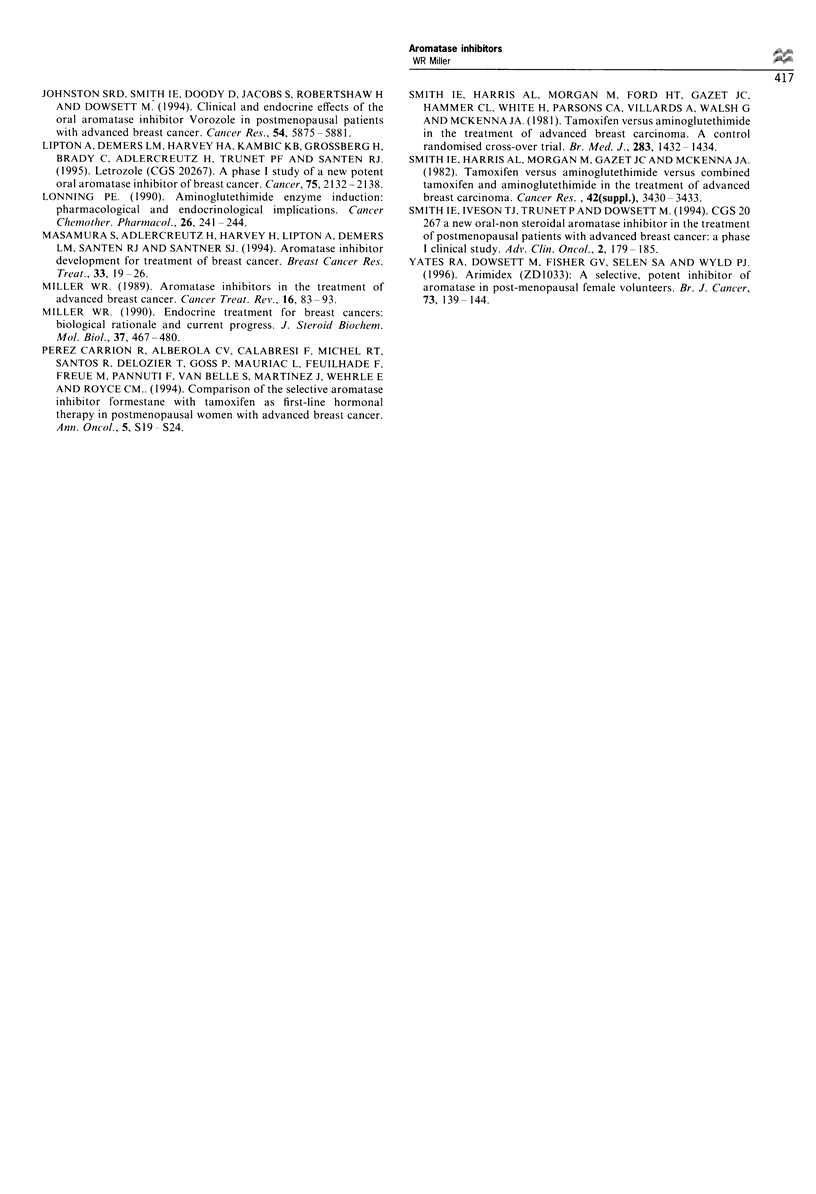# Aromatase inhibitors--where are we now?

**DOI:** 10.1038/bjc.1996.73

**Published:** 1996-02

**Authors:** W. R. Miller

**Affiliations:** University Department of Clinical Oncology, Western General Hospital, Edinburgh, UK.


British Journal of Cancer (1996) 73, 415-417
© 1996 Stockton Press All rights reserved 0007-0920/96 $12.00

GUEST EDITORIAL

### Aromatase inhibitors – where are we now?

WR Miller

*ICRF Medical Oncology Unit, University Department of Clinical Oncology, Western General Hospital, Edinburgh EH4 2XU, UK.*

**Keywords:** aromatase inhibitor; breast cancer

The rationale behind endocrine therapy for breast cancer is the knowledge that certain tumours require oestrogen for their continued growth. Sources of oestrogen differ according to menopausal status; ovarian production predominates in premenopausal women whereas synthesis in peripheral tissues such as fat, muscle and the tumour itself is more important in post-menopausal patients (Miller, 1990). The use of drugs specifically designed to block oestrogen biosynthesis irrespective of site of production is therefore an attractive strategy. Oestrogens lie at the end of a multistep pathway. Blockade can be achieved by inhibiting any of the individual transformations but more specific suppression is achieved by inhibiting the final step, which is unique to oestrogen biosynthesis. This reaction converts androgens into oestrogens by creating an aromatic ring in the steroid molecule (hence the trivial name for the enzyme of 'aromatase'). Consequently, enormous efforts have been expended in the development of aromatase inhibitors by synthesising either substrate analogues or drugs that interfere with the enzyme's prosthetic cytochrome p450 group.

It has been known for some time that drug-induced inhibition of the aromatase enzyme may produce therapeutic benefits in patients with breast cancer. Agents such as aminoglutethimide were used without initially realising that they had anti-aromatase properties. Nevertheless, the major benefits of aminoglutethimide (which include a 33% objective response rate in unselected post-menopausal patients with advanced breast cancer) are probably achieved through inhibition of the aromatase system (Miller, 1989). However, aminoglutethimide is not a potent aromatase inhibitor; it also lacks specificity and side-effects may be produced that are unrelated to oestrogen deprivation. Considerable resources have been invested in the development of second- and third-generation drugs (Combs *et al.*, 1995). Results of studies on these aromatase inhibitors are now being published, as is reflected by the current issue of the *Br. J. Cancer*, which contains two such articles (Yates *et al.*, 1996 and Bonnefoi *et al.*, 1996). It is thus opportune to review the current status of these drugs in terms of (i) anti-aromatase and endocrinological effects, (ii) clinical tolerability and efficacy, (iii) relationship with established endocrine treatments, (iv) future applications and (v) theoretical and practical perspectives.

### Anti-aromatase and endocrinological effects

Among the drugs under current scrutiny are steroidal analogues such as formestane and exemestane and non-steroidals such as fadrozole, vorozole, letrozole and Arimidex. All are substantially more potent than aminoglutethimide as inhibitors of the aromatase enzyme (see Table I). This potential is reflected by *in vivo* effects on circulating oestrogens. For example in this current issue Yates *et al.* report that small doses of Arimidex suppress oestradiol levels by about 80% and, in a number of subjects, values fell below limits of detection. These effects were achieved without significant influences on other classes of steroid hormones. Similar results have been reported by others for letrozole (Demers, 1994; Lipton *et al.*, 1995), and vorozole (Johnston *et al.*, 1994; Goss *et al.*, 1995). Of the new non-steroidal aromatase inhibitors, fadrozole seems less effective in both inhibiting aromatase and suppressing circulating oestrogens (Demers, 1994). Doubts have also been expressed about its specificity; changes in aldosterone secretion have been reported (Demers *et al.*, 1993), but at doses that produce maximal suppression of oestrogen, effects on aldosterone may not be of clinical significance (Dowsett *et al.*, 1994).

### Clinical tolerability and efficacy

These new aromatase inhibitors are administered orally (the exception is formestane, which requires intramuscular injection) and appear to be remarkably well tolerated, with no greater incidence of side-effects than might be expected from a placebo or from oestrogen suppression. However, it should be noted that the duration of treatment in most patients is still extremely limited.

Despite the drugs being initially used in heavily pretreated patients with advanced disease, anti-tumour effects are encouraging. Formestane has been associated with an objective response rate of 33% and remissions have been seen in patients previously treated with aminoglutethimide (Coombes, 1989). This issue includes a report that fadrozole produces a 17% objective response rate in recurrent breast cancer after tamoxifen failure (Bonnefoi *et al.*, 1996). Similar observations in tamoxifen-resistant disease have been made for vorozole, with Johnston *et al.* (1994) reporting a 33% response rate and Goss *et al.* (1995) a 17% rate. Early data on letrozole also indicate that beneficial tumour remissions may be achieved in patients resistant to other endocrine and chemotherapeutic manoeuvres (Smith *et al.*, 1994). Given this promise it is essential that direct comparative studies are performed against established endocrine therapies. Clinical trials of primary treatment are underway and their results are

**Table I** Relative *in vitro* potency of aromatase inhibitors as determined using placental microsomes as a test system

| | |
|---|---|
| Aminoglutethimide | 1 |
| Formestane | 60 |
| Exemestane | 60 |
| Fadrozole | 380 |
| Arimidex | 200 |
| Vorozole | 1000 |
| Letrozole | 200 |

Received 19 September 1995; accepted 6 October 1995

eagerly awaited. Interestingly, a comparison of formestane and tamoxifen as primary treatment found similar response rates with both drugs (Perez-Carrion *et al.*, 1994).

### Relationship with established endocrine treatments

That responses have been achieved with the new aromatase inhibitors following treatment failure with antioestrogens or less powerful aromatase inhibitors suggests that they warrant a place as second-line endocrine therapies. Whether they can replace tamoxifen as a first-line therapy in all or some patients depends upon the results of on-going trials. Even if response rates and toxicities are similar to those of tamoxifen, there may be a lesson to be learnt from the experience with aminoglutethimide. Thus, response rates to first-line therapy with tamoxifen and aminoglutethimide are similar but, whereas aminoglutethimide is effective in about 30% of patients when given as second-line therapy to tamoxifen, the antioestrogen less frequently causes remission after aminoglutethimide (Smith *et al.*, 1981), which dictates a logical sequence of tamoxifen followed by aminoglutethimide. Whether this phenomenon will apply to other more specific aromatase inhibitors is unknown. (One aspect of aminoglutethimide's lack of specificity may be to enhance drug metabolism; Lonning, 1990). Similarly, the disappointing results obtained when aminoglutethimide is combined with other endocrine procedures (Smith *et al.*, 1982) should not deter the use of combination therapies based around newer aromatase inhibitors. The concept of using potent antioestrogens in tandem with equally potent aromatase inhibitors to achieve total oestrogen blockade may yet prove irresistible (but see below).

### Future applications

The potent and specific characteristics of the new aromatase inhibitors suggest that they may have a wider utility than previous drugs. For example, it has always been puzzling as to why aromatase inhibitors should be effective after failure to antioestrogen if both types of drug have a common mechanism of oestrogen deprivation but the commonly expounded reasons for this are (i) antioestrogens such as tamoxifen are partial oestrogen agonists and may compete ineffectively for oestrogen receptors or (ii) under the selective pressure of antioestrogen treatment tumours become increasingly sensitive to oestrogen. In these circumstances aromatase inhibitors that reduce oestrogen levels may produce antitumour effects. If this is the case, more potent aromatase inhibitors that suppress oestrogen levels beyond those previously achievable, could increase cell kill and produce higher response rates.

A second area for exploitation is as an adjuvant to surgery in early stages of the disease. The acceptability of adjuvant therapy depends critically upon lack of side-effects. This is especially important for adjuvant endocrine therapy, which probably needs to be given over an extended time period for most beneficial effects. Because, in comparison with previous aromatase inhibitors, second- and third-generation drugs appear to lack toxicity, there is pressure for adjuvant use. However, this may be premature until results are available on long-term administration. The concern is that prolonged suppression of oestradiol to unassayable levels may have severe detrimental effects on bone and the vasculature.

More potent aromatase inhibitors may also be effective in situations in which aromatase activity is high or induced. For example aminoglutethimide is not effective in premenopausal women (Harris *et al.*, 1982), presumably because it cannot inhibit the inherently high aromatase activity in the ovary or the reflex feedback loops that result in compensatory increases in enzyme and androgen substrate. More potent and specific inhibitors may be able to be given in sufficient doses to overcome these effects and suppress oestrogens to post-menopausal levels.

### Theoretical and practical perspectives

There are theoretical reasons as to why specific aromatase inhibitors may not achieve complete oestrogenic blockade *in vivo*. Thus, whereas the drugs inhibit peripheral aromatase almost completely, levels of circulating oestrogens fall only by 40–85% (Masamura *et al.*, 1994). Specific aromatase inhibitors, even if totally effective, will not effect (1) the synthesis of androgens such as Δ 5-androstenediol, which are capable of oestrogenic effects (Hackenberg *et al.*, 1993), nor (2) the action of exogenous oestrogens such as dietary phyto-oestrogens and industrial contaminants such as pesticides and plasticisers (which may act as weak oestrogens). Although controversial, it is possible that these alternative oestrogenic sources may maintain hormone-dependent tumour growth. In these circumstances, pure antioestrogens have greater versatility in that they should block trophic effects irrespective of the source of oestrogen. However, potent specific aromatase inhibitors are powerful tools for research and their use is likely to yield fundamental information about aromatase activity and the diverse sources of oestrogens.

Finally there is the practical consideration as to which of the new inhibitors will make the greatest clinical impact. At present, this is impossible to answer. Several inhibitors have similar profiles with regard to their potency, specificity, clinical efficacy and tolerability. It may come down to cost and marketing – a chastening thought given the vast scientific/clinical effort invested in developing and assessing the drugs.

### References

BONNEFOI HR, SMITH IE, DOWSETT M, TRUNET PF, HOUSTON SJ, DA LUZ RJ, RUBENS RD, COOMBES RC AND POWLES TJ. (1996). Therapeutic effects of the aromatase inhibitor fadrozole hydrochloride in advanced breast cancer. *Br. J. Cancer*, **73**, 135–138.

COMBS DW. (1995). Recent developments in aromatase inhibitors. *Expert Opin. Ther. Patents*, **5**, 529–534.

COOMBES RC, STEIN RC AND DOWSETT M. (1989). Aromatase inhibitors in human breast cancer. *Proc. R. Soc. Edin.*, **95B**, 293–304.

DEMERS LM. (1984). Effects of Fadrozole (CGS 16949A) and Letrozole (CGS 20267) on the inhibition of aromatase activity in breast cancer patients. *Breast Cancer Res. Treat.*, **30**, 95–102.

DEMERS LM, LIPTON A, HARVEY HA, HANAGHAN J, MALAGHA M AND SANTEN RJ. (1993). The effects of long term fadrozole hydrochloride treatment in patients with advanced stage breast cancer. *J. Steroid Biochem. Mol. Biol.*, **44**, 683–685.

DOWSETT M, SMITHERS D, MOORE J, TRUNET PF, COOMBES RC, POWLES TJ, RUBENS R AND SMITH IE. (1994). Endocrine changes with the aromatase inhibitor fadrozole hydrochloride in breast cancer. *Eur. J. Cancer*, **30A**, 1453–1458.

GOSS PE, CLARK RM, AMBUS U, WEIZEL HAE, WADDEN NA, CRUMP M, WALDE D, TYE LM, DE COSTER R AND BRUYNSEELS J. (1995). Phase II study of vorozole (R83842), a new aromatase inhibitor, in postmenopausal women with advanced breast cancer in progression on tamoxifen. *Clin. Cancer Res.*, **1**, 287–294.

HACKENBERG R, TURGETTO I, FILMER A AND SCHULZ KD. (1993). Estrogen and androgen receptor-mediated stimulation and inhibition of proliferation by androst-5-ene-3-beta, 17-beta-diol in human mammary cancer cells. *J. Steroid Biochem.*, **46**, 597–603.

HARRIS AL, DOWSETT M, JEFFCOATE SL, MCKENNA JA, MORGAN M AND SMITH IE. (1982). Endocrine and therapeutic effects of aminoglutethimide in premenopausal patients with breast cancer. *J. Clin. Endocr. Metab.*, **55**, 718–720.

JOHNSTON SRD, SMITH IE, DOODY D, JACOBS S, ROBERTSHAW H AND DOWSETT M. (1994). Clinical and endocrine effects of the oral aromatase inhibitor Vorozole in postmenopausal patients with advanced breast cancer. *Cancer Res.*, **54**, 5875–5881.

LIPTON A, DEMERS LM, HARVEY HA, KAMBIC KB, GROSSBERG H, BRADY C, ADLERCREUTZ H, TRUNET PF AND SANTEN RJ. (1995). Letrozole (CGS 20267). A phase I study of a new potent oral aromatase inhibitor of breast cancer. *Cancer*, **75**, 2132–2138.

LONNING PE. (1990). Aminoglutethimide enzyme induction: pharmacological and endocrinological implications. *Cancer Chemother. Pharmacol.*, **26**, 241–244.

MASAMURA S, ADLERCREUTZ H, HARVEY H, LIPTON A, DEMERS LM, SANTEN RJ AND SANTNER SJ. (1994). Aromatase inhibitor development for treatment of breast cancer. *Breast Cancer Res. Treat.*, **33**, 19–26.

MILLER WR. (1989). Aromatase inhibitors in the treatment of advanced breast cancer. *Cancer Treat. Rev.*, **16**, 83–93.

MILLER WR. (1990). Endocrine treatment for breast cancers: biological rationale and current progress. *J. Steroid Biochem. Mol. Biol.*, **37**, 467–480.

PEREZ CARRION R, ALBEROLA CV, CALABRESI F, MICHEL RT, SANTOS R, DELOZIER T, GOSS P, MAURIAC L, FEUILHADE F, FREUE M, PANNUTI F, VAN BELLE S, MARTINEZ J, WEHRLE E AND ROYCE CM.. (1994). Comparison of the selective aromatase inhibitor formestane with tamoxifen as first-line hormonal therapy in postmenopausal women with advanced breast cancer. *Ann. Oncol.*, **5**, S19–S24.

SMITH IE, HARRIS AL, MORGAN M, FORD HT, GAZET JC, HAMMER CL, WHITE H, PARSONS CA, VILLARDS A, WALSH G AND MCKENNA JA. (1981). Tamoxifen versus aminoglutethimide in the treatment of advanced breast carcinoma. A control randomised cross-over trial. *Br. Med. J.*, **283**, 1432–1434.

SMITH IE, HARRIS AL, MORGAN M, GAZET JC AND MCKENNA JA. (1982). Tamoxifen versus aminoglutethimide versus combined tamoxifen and aminoglutethimide in the treatment of advanced breast carcinoma. *Cancer Res.* , **42(suppl.)**, 3430–3433.

SMITH IE, IVESON TJ, TRUNET P AND DOWSETT M. (1994). CGS 20 267 a new oral-non steroidal aromatase inhibitor in the treatment of postmenopausal patients with advanced breast cancer: a phase I clinical study. *Adv. Clin. Oncol.*, **2**, 179–185.

YATES RA, DOWSETT M, FISHER GV, SELEN SA AND WYLD PJ. (1996). Arimidex (ZD1033): A selective, potent inhibitor of aromatase in post-menopausal female volunteers. *Br. J. Cancer*, **73**, 139–144.